# High implantation of a balloon-expandable valve above the left ventricular outflow calcification improves the prosthetic valve function without increasing complications: a case series

**DOI:** 10.1093/ehjcr/ytaf007

**Published:** 2025-01-10

**Authors:** Kyohei Onishi, Kazuki Mizutani, Naoko Soejima, Kosuke Fujita, Masakazu Yasuda, Masafumi Ueno, Genichi Sakaguchi, Gaku Nakazawa

**Affiliations:** Division of Cardiology, Department of Medicine, Kindai University Faculty of Medicine, 377-2 Ohno-Higashi, Osakasayama, Osaka 589-8511, Japan; Division of Cardiology, Department of Medicine, Kindai University Faculty of Medicine, 377-2 Ohno-Higashi, Osakasayama, Osaka 589-8511, Japan; Division of Cardiology, Department of Medicine, Kindai University Faculty of Medicine, 377-2 Ohno-Higashi, Osakasayama, Osaka 589-8511, Japan; Division of Cardiology, Department of Medicine, Kindai University Faculty of Medicine, 377-2 Ohno-Higashi, Osakasayama, Osaka 589-8511, Japan; Division of Cardiology, Sakurabashi Watanabe Hospital, 4-3-51 nakanoshima kita-ku, Osaka 530-0005, Japan; Division of Cardiology, Department of Medicine, Kindai University Faculty of Medicine, 377-2 Ohno-Higashi, Osakasayama, Osaka 589-8511, Japan; Division of Cardiovascular Surgery, Department of Medicine, Kindai University Faculty of Medicine, 377-2 Ohno-Higashi, Osakasayama, Osaka 589-8511, Japan; Division of Cardiology, Department of Medicine, Kindai University Faculty of Medicine, 377-2 Ohno-Higashi, Osakasayama, Osaka 589-8511, Japan

**Keywords:** Annular rupture, Aortic stenosis, Left ventricular outflow tract calcification, Paravalvular leakage, Transcatheter aortic valve replacement, Case report

## Abstract

**Background:**

The initial outcomes of transcatheter aortic valve replacement in patients with left ventricular outflow tract calcification are poor. Furthermore, balloon-expandable transcatheter aortic valve replacement is associated with an increased risk of annular rupture, and self-expandable transcatheter aortic valve replacement is associated with worse post-operative residual paravalvular leakage grades. Therefore, developing an optimal method for transcatheter aortic valve replacement for patients with left ventricular outflow tract calcification is desirable.

**Case summary:**

We present two cases of successful balloon-expandable transcatheter aortic valve replacement, wherein the transcatheter heart valve was implanted above the left ventricular outflow tract calcification to avoid annular rupture and paravalvular leakage, and one case each of balloon-expandable and self-expandable transcatheter aortic valve replacements, wherein the transcatheter heart valve was implanted at a normal height. Although annular rupture did not occur in any of the cases, more-than-mild paravalvular leakage persisted post-operatively in cases where the transcatheter heart valve was placed at a normal height.

**Discussion:**

Annular rupture is more likely to occur in areas with high calcification at the joint than in noncalcified areas. Furthermore, the greater the calcification in the landing zone of the transcatheter heart valve, the more the paravalvular leakage persists. Therefore, high implantation of transcatheter heart valves above the left ventricular outflow tract calcification can be an effective method to avoid annular rupture and paravalvular leakage.

**Learning points**

- The initial outcomes of transcatheter aortic valve replacement (TAVR) in patients with severe left ventricular outflow tract calcification (LVOTC) are poor; therefore, establishing an effective TAVR technique is desirable.
- High implantation of the balloon-expandable valve above the LVOTC avoids interference with the calcification and improves transcatheter heart valve crimping, thus reducing the risk of annular rupture and residual paravalvular leakage.

## Introduction

Transcatheter aortic valve replacement (TAVR) is an alternative treatment option for patients with severe aortic stenosis (AS). Transcatheter aortic valve replacement is comparable to surgical AVR, and the excellent short- and long-term outcomes have expanded its application to patients at any surgical risk.^1–3^ Furthermore, a comparison of the clinical results of different transcatheter heart valve (THV) types has recently been reported, such as the possibility that self-expandable TAVR (SE-TAVR) may have a lower incidence of bioprosthetic valve dysfunction than balloon-expandable TAVR (BE-TAVR), especially in patients with a small annulus.^4^ However, unresolved issues remain with TAVR. In particular, the initial outcomes of TAVR in patients with left ventricular outflow tract calcification (LVOTC) are poor. Furthermore, BE-TAVR is associated with an increased risk of annular rupture, and SE-TAVR is associated with a higher post-operative residual paravalvular leakage (PVL) grade.^5,6^ Therefore, the development of a novel and efficient TAVR strategy for patients with LVOTC is desirable. In this case series, we demonstrate the potential of high implantation of the BE valve (BEV) above the LVOTC as an effective method compared with the implantation of BEV and SE valve (SEV) at a normal height.

## Summary figure

Diagram of valves to be used and implantation depth.

**Figure:**
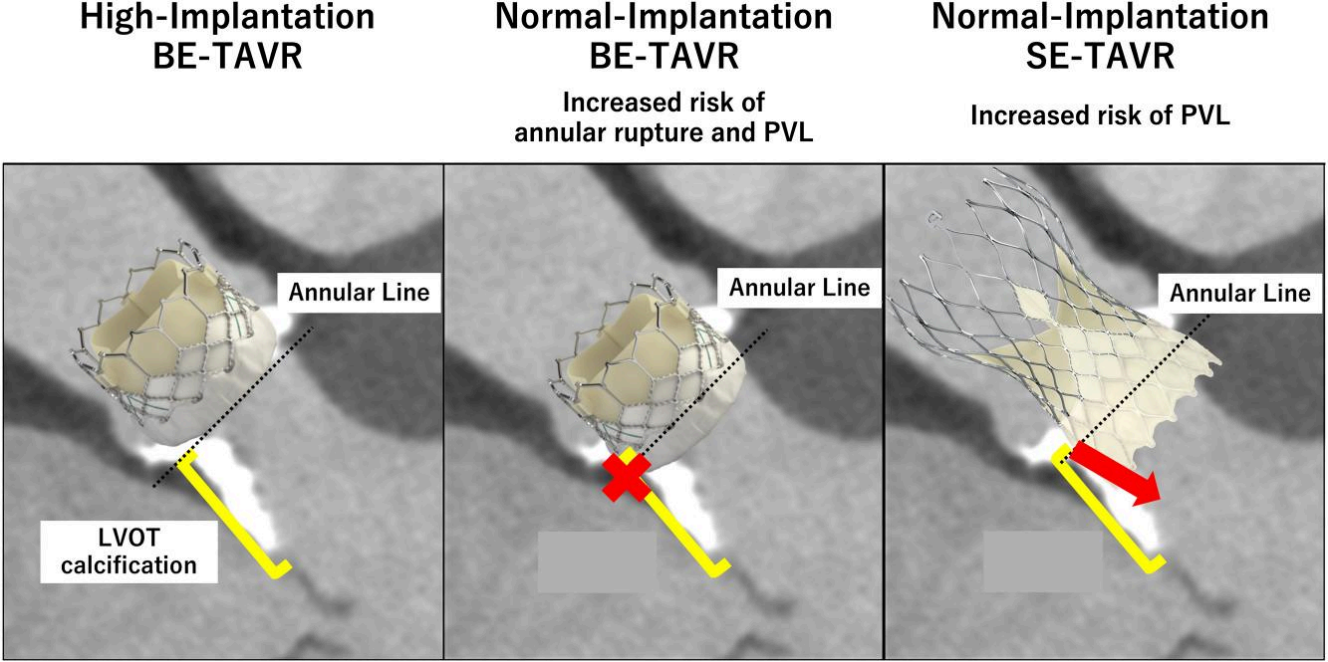


## Case presentation

### Patient 1

**Figure 1:**
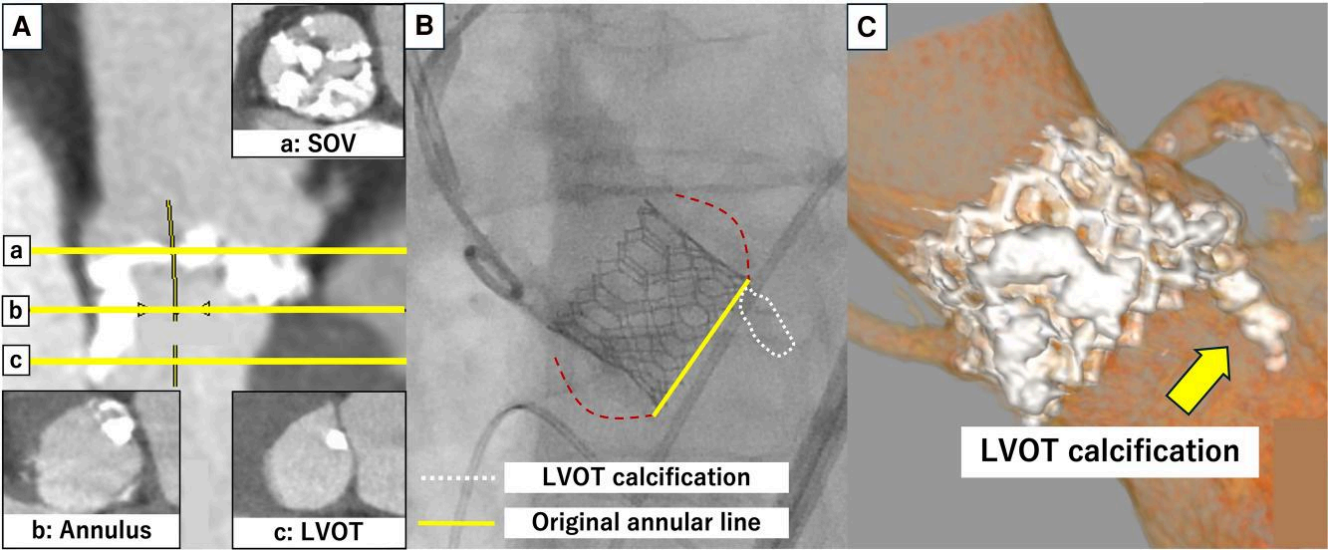
Pre- and post-procedural images of Case 1. (*A*) Pre-procedural computed tomography: a, sinus of Valsalva level; b, annular level; c, left ventricular outflow tract level. (*B*) Aortography after transcatheter aortic valve replacement. (*C*) Post-procedural computed tomography.

An 88-year-old woman with no prior history of cardiovascular disease was transferred to our institution for TAVR because of decompensated heart failure secondary to severe AS. The vital signs at admission were temperature 36.8°C, blood pressure 93/53 mmHg, heart rate 58 b.p.m., and oxygen saturation 96% in room air. Transthoracic echocardiography (TTE) revealed very severe AS, with a mean aortic valve pressure gradient (mAVPG) of 92 mmHg, an aortic valve peak flow velocity (AVPFV) of 6.4 m/s, an aortic valve area (AVA) of 0.44 mm^2^, and an ejection fraction (EF) of 57%. Electrocardiogram (ECG) showed no bundle branch block. Multislice computed tomography (MSCT) showed that the annulus had an area of 405.1 mm^2^, a mean diameter of 23.6 mm, left coronary artery (LCA) height of 15.0 mm, right coronary artery (RCA) height of 16.0 mm, bulky calcification on all three leaflets, severe LVOTC [maximum diameter: 11.4 mm, calcium volume: 126 mm³ using 850 Hounsfield units (HU)] below the left coronary cusp (LCC) (*Figure 1*), and membranous septum (MS) length of 1.6 mm. We performed TAVR with high implantation above the LVOTC using a 23 mm SAPIEN3 (Edwards Lifesciences) via the transfemoral approach. The final aortogram showed that SAPIEN3 was implanted at a level higher than the original annular line, and the PVL grade was trace. The patient’s recovery was uneventful following the procedure. Post-operative ECG showed no complications of conduction disturbances. Transthoracic echocardiography showed a good valve function with a trivial PVL, an mAVPG of 11.6 mmHg, and an effective orifice area index (EOAi) of 1.37 cm^2^/m^2^ at discharge. Multislice computed tomography performed 1 month after the procedure revealed that SAPIEN3 had expanded into a regular circle and was implanted above the LVOTC.

### Patient 2

**Figure 2:**
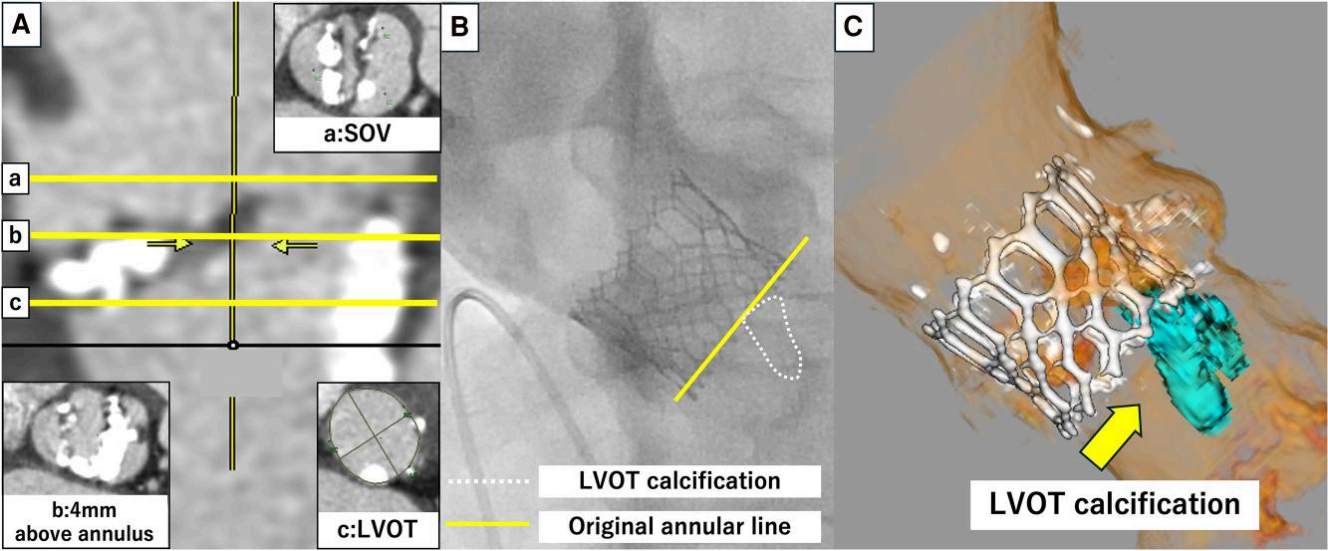
Pre- and post-procedural images of Case 2. (*A*) Pre-procedural computed tomography: a, sinus of Valsalva level; b, 4 mm above the annular level; c, left ventricular outflow tract level. (*B*) Aortography after transcatheter aortic valve replacement. (*C*) Post-procedural computed tomography.

A 79-year-old man with hypertension and dyslipidaemia on medication was admitted to our hospital for orthopnoea. The vital signs at admission were temperature 36.9°C, blood pressure 109/42 mmHg, heart rate 72 b.p.m., and oxygen saturation 97% under 5 L of oxygen mask. Transthoracic echocardiography revealed a bicuspid aortic valve and severe AS with an mAVPG of 45.6 mmHg, an AVPFV of 4.8 m/s, an AVA of 0.4 mm^2^, and an EF of 50%. Electrocardiogram showed no bundle branch block. Multislice computed tomography showed type-1 (R–L) bicuspid aortic valve. The annulus had an area of 472.6 mm^2^, a mean diameter of 24.9 mm (diameter of the intra-raphe: 21.1 mm × 25.7 mm), an LCA height of 16.7 mm, an RCA height of 15.9 mm, bulky calcification on both leaflets, severe LVOTC (maximum diameter: 12.1 mm, calcium volume: 68 mm³ using 850 HU) below the side of the LCA (*Figure 2*), and an MS length of 1.3 mm. We performed emergency transfemoral TAVR with high implantation above the LVOTC by using a 23 mm SAPIEN3. The final aortogram showed that SAPIEN3 was implanted at a level higher than the original annular line. The heart failure condition stabilized following the procedure. The patient was transferred to the general ward 6 days after TAVR and subsequently discharged. Post-operative ECG showed no complications of conduction disturbances. Transthoracic echocardiography showed a favourable function of the TAV prosthesis without PVL, an mAVPG of 9 mmHg, and an EOAi of 1.07 cm^2^/m^2^ at discharge. Multislice computed tomography performed 1 month after TAVR revealed that SAPIEN3 was implanted above the LVOTC.

### Patient 3

**Figure 3:**
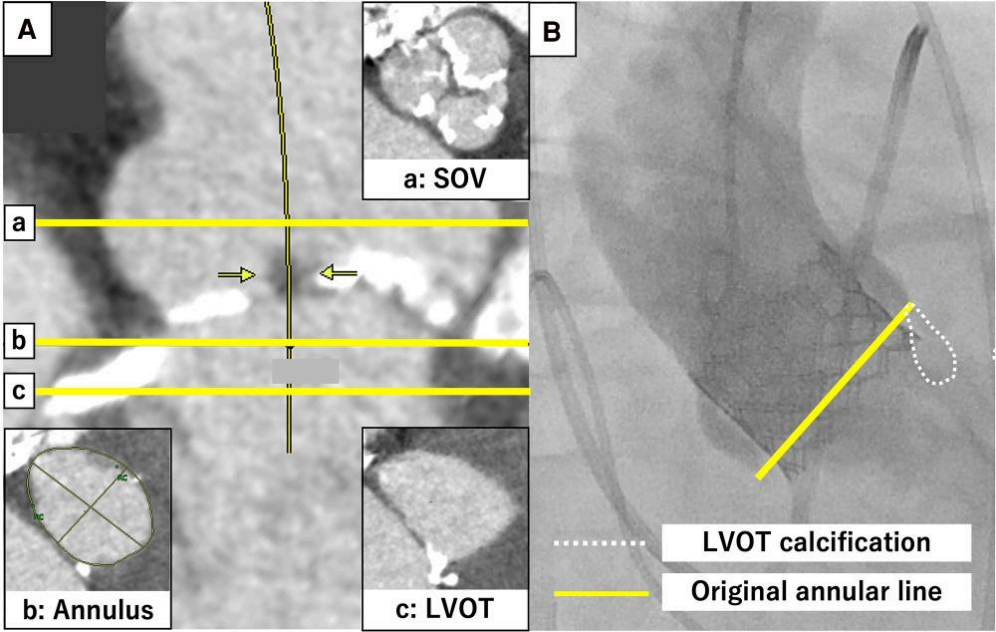
Pre- and post-procedural images of Case 3. (*A*) Pre-procedural computed tomography: a, sinus of Valsalva level; b, annular level; c, left ventricular outflow tract level. (*B*) Aortography after transcatheter aortic valve replacement.

An 88-year-old woman with a history of hypertension, dyslipidaemia, and arteriosclerosis obliterans was transferred to our institution for the management of intractable heart failure secondary to severe AS. The vital signs at admission were temperature 37.2°C, blood pressure 102/79 mmHg, heart rate 94 b.p.m., and oxygen saturation 98% under 8 L of oxygen mask. Transthoracic echocardiography revealed low-flow and low-gradient severe AS with an mAVPG of 29.7 mmHg, an AVPFV of 3.7 m/s, an AVA of 0.53 mm^2^, and left ventricular dysfunction (EF: 20%). The patient experienced cardiogenic shock owing to severe AS. Despite providing treatment with inotropic agents and intra-aortic balloon pumping (IABP), the condition of heart failure remained unstable. The decision to perform emergency TAVR was made at a heart team conference. Electrocardiogram showed no bundle branch block. Multislice computed tomography showed that the annulus had an area of 516.6 mm^2^, a mean diameter of 26.5 mm, an LCA height of 17.6 mm, an RCA height of 19.6 mm, calcification on all three leaflets, severe LVOTC (maximum diameter: 12.1 mm, calcium volume: 21 mm³ using 850 HU) below the LCC (*Figure 3*), and an MS length of 3.0 mm. Clinical Frailty Scale (CFS) was 6, and the Society of Thoracic Surgery Risk Score (STS Score) predicted risk of mortality score was 18.2%.

We performed TAVR conventionally using a 26 mm SAPIEN3 (Edwards Lifesciences) through the transfemoral approach with extracorporeal membrane oxygenation (ECMO) support. After valve deployment, aortography revealed severe PVL. Balloon post-dilation was performed twice with a SAPIEN balloon (+2 and +4 mL). The final aortogram showed that PVL decreased to a moderate grade. Although PVL remained mild to moderate after TAVR, the haemodynamics stabilized. Extracorporeal membrane oxygenation was withdrawn intraoperatively, the IABP was removed the next day, and inotropic agents were successfully weaned off 2 days after the procedure. The patient was subsequently adjusted for medications and transferred to a rehabilitation hospital. Post-operative ECG showed no complications of conduction disturbances. Echocardiography revealed mild-to-moderate PVL, an mAVPG of 4 mmHg, and an EOAi of 1.51 cm^2^ at discharge.

We inferred that, even with the implantation of the BEV, dilatation at the conventional height caused poor dilatation owing to severe LVOTC, which led to persistent PVL.

### Patient 4

**Figure 4:**
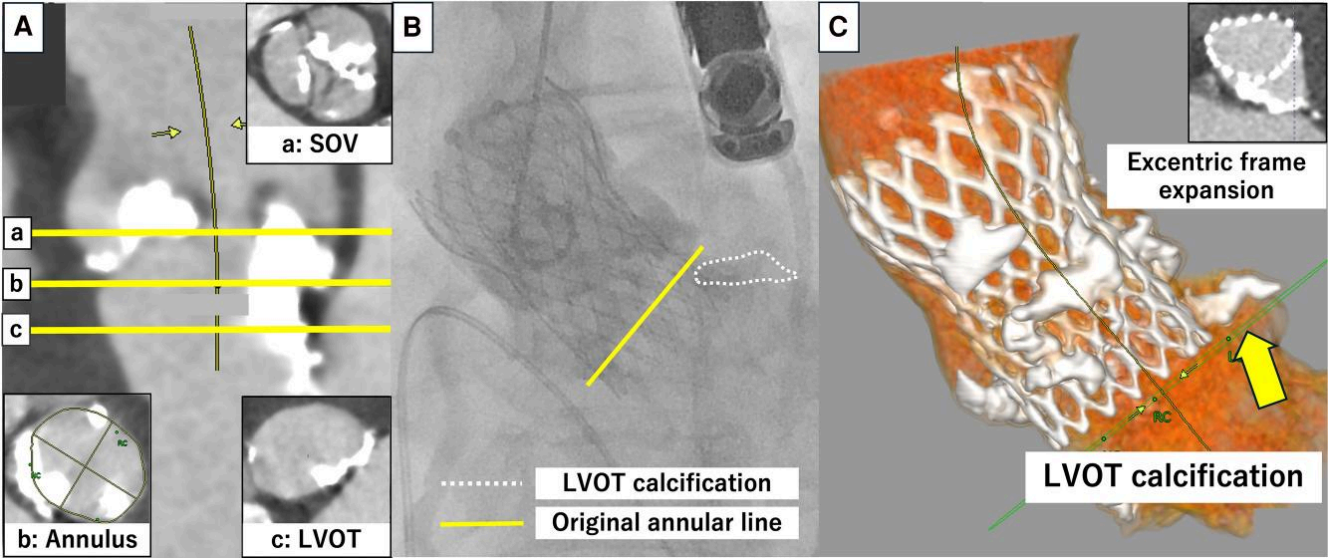
Pre- and post-procedural images of Case 4. (*A*) Pre-procedural computed tomography: a, sinus of Valsalva level; b, annular level; c, left ventricular outflow tract level. (*B*) Aortography after transcatheter aortic valve replacement. (*C*) Post-procedural computed tomography.

An 84-year-old woman with no prior history of cardiovascular disease presented with shortness of breath. She was diagnosed with severe AS based on an mAVPG of 60 mmHg, an AVPFV of 4.9 m/s, an AVA of 0.53 mm^2^, and an EF of 53%, and was admitted to our hospital on the same day. The vital signs at admission were temperature 37.0°C, blood pressure 122/83 mmHg, heart rate 85 b.p.m., and oxygen saturation 100% under 2 L of nasal cannula. Electrocardiogram showed no bundle branch block. Multislice computed tomography showed that the annulus had an area of 425.2 mm^2^, a mean diameter of 24.9 mm, a perimeter of 77.0 mm, an LCA height of 15.0 mm, an RCA height of 16.0 mm, bulky calcification on each of the three leaflets, severe LVOTC (maximum diameter: 10.5 mm, calcium volume: 58 mm³ using 850 HU) below the LCC (*Figure 4*), and an MS length of 3.7 mm. Trans-subclavian TAVR was performed using a 26 mm Evolut Pro+ (Medtronic) due to calcification and stenosis in the bilateral iliac arteries. The valve depth was 5.8 mm, and aortography revealed severe PVL. Balloon post-dilation with a 25 mm Z-MED (JMS) was partially effective, and the final PVL grade was moderate. Post-operative ECG showed no complications of conduction disturbances. Transthoracic echocardiography showed mild-to-moderate PVL, an mAVPG of 9 mmHg, and an EOAi of 1.3 cm^2^/m^2^ at discharge. Multislice computed tomography performed 1 month after TAVR revealed eccentric expansion of the Evolut owing to severe calcification.

## Discussion

In this case series, we present two cases of BE-TAVR performed using the high-implantation technique to place a THV deployed above the LVOTC (detailed instruction can be found in the Supplementary material) and one case each of BE-TAVR and SE-TAVR, wherein the THV was implanted at a normal height. A previous report showed that annular rupture occurred more frequently in patients with moderate or severe LVOTC than in those without LVOTC (2.3% vs. 0.2%; *P* < 0.001), especially in patients treated with older-generation BEVs (SAPIEN XT).^6^ Furthermore, moderate or severe LVOTC resulted in more residual PVL after TAVR regardless of the type of the valve used, and the residual PVL grade was worse with SE-TAVR than with BE-TAVR.^6^ In Cases 3 (BE-TAVR) and 4 (SE-TAVR) of this study, the PVL grade remained approximately moderate as a result of THV implantation at a normal height even though a newer-generation device (SAPIEN3 and EvolutPro) was used. The mechanism of annular rupture is similar to that of coronary artery perforation in percutaneous coronary intervention and is more likely to occur in areas of high calcification at the joint than in noncalcified areas.^7,8^ Furthermore, a previous study reported that the more calcification in the landing zone of the THV, the more the PVL remains.^9^ Therefore, a higher placement of THVs above the LVOTC, as in Cases 1 and 2 of this study, may prevent annular rupture and significant PVL, even if a BEV is used. As pre-operative right bundle branch block, short MS length, and LVOTC have all been reported to be risk factors for conduction disturbance after TAVR, high-implantation THV can potentially reduce post-operative conduction disturbances and the need for pacemaker implantation.^10,11^ On the other hand, we should consider the risk of valve embolization and coronary obstruction due to the high-implantation technique. Although patients with severe LVOTC often have huge leaflet calcification and fixation by THV expansion is easy and embolization is unlikely to occur, it is important to further reduce the risk of valve embolization by slow inflation while implanting a THV. Most causes of coronary obstruction have been reported to be due to native valve compression or due to the inner skirt of the THV.^12^ The BE-THV inner skirt height is 7.9–11.6 mm (data disclosed by Edwards Lifesciences), and it is difficult to adjust commissure alignment in BE-TAVR. If the height from the annular plane to the coronary ostium is lower than the inner skirt height, the risk of coronary obstruction is high, and we believe that high implantation above the LVOTC is difficult. Furthermore, we can expect a similar efficacy if SEVs are implanted above the LVOTC; however, high implantation of SEVs is expected to render coronary access difficult and cause sinus sequestration.^10^ Even with BE-TAVR, the feasibility of coronary access after first TAVR and future TAV in TAV procedures should be considered with lifetime management in mind.^13^

## Conclusion

We conclude that high implantation of BEVs above the LVOTC has the potential to improve the PVL grade without increasing the risk of complications.

## Supplementary Material

ytaf007_Supplementary_Data

## Data Availability

Data sharing is not applicable to this article, as no data sets were generated or analysed during the current study. The data underlying this article are available in the article and online supplementary material.

## Lead author biography

Dr Onishi graduated from Kindai University Hospital in 2015. He is working at Kindai University Hospital, Department of Cardiology. His speciality is ischaemic heart disease and structural heart disease.

## References

[1] Leon MB , Smith CR, Mack M, Miller DC, Moses JW, Svensson LG, et al Transcatheter aortic-valve implantation for aortic stenosis in patients who cannot undergo surgery. N Engl J Med 2010;363:1597–1607. 20961243 10.1056/NEJMoa1008232

[2] Leon MB , Smith CR, Mack MJ, Makkar RR, Svensson LG, Kodali SK, et al Transcatheter or surgical aortic-valve replacement in intermediate-risk patients. N Engl J Med 2016;374:1609–1620. 27040324 10.1056/NEJMoa1514616

[3] Mack MJ , Leon MB, Thourani VH, Pibarot P, Hahn RT, Genereux P, et al Transcatheter aortic-valve replacement in low-risk patients at five years. N Engl J Med 2023;389:1949–1960. 37874020 10.1056/NEJMoa2307447

[4] Herrmann HC , Mehran R, Blackman DJ, Bailey S, Möllmann H, Abdel-Wahab M, et al Self-expanding or balloon-expandable TAVR in patients with a small aortic annulus. N Engl J Med 2024;390:1959–1971. 38587261 10.1056/NEJMoa2312573

[5] Sá MP , Van den Eynde J, Malin JH, Torregrossa G, Sicouri S, Ramlawi B. Impact of left ventricle outflow tract calcification on the outcomes of transcatheter aortic valve implantation: a study-level meta-analysis. J Card Surg 2022;37:1379–1390. 35152472 10.1111/jocs.16306

[6] Okuno T , Asami M, Heg D, Lanz J, Praz F, Hagemeyer D, et al Impact of left ventricular outflow tract calcification on procedural outcomes after transcatheter aortic valve replacement. JACC Cardiovasc Interv 2020;13:1789–1799. 32763071 10.1016/j.jcin.2020.04.015

[7] Hendry C , Fraser D, Eichhofer J, Mamas MA, Fath-Ordoubadi F, El-Omar M, et al Coronary perforation in the drug-eluting stent era: incidence, risk factors, management and outcome: the UK experience. EuroIntervention 2012;8:79–86. 22580251 10.4244/EIJV8I1A13

[8] Avula V , Karacsonyi J, Kostantinis S, Simsek B, Rangan BV, Gutierrez AA, et al Incidence, treatment, and outcomes of coronary artery perforation during percutaneous coronary intervention. J Invasive Cardiol 2022;34:E499–E504. 35714223 10.25270/jic/21.00358

[9] Mauri V , Frohn T, Deuschl F, Mohemed K, Kuhr K, Reimann A, et al Impact of device landing zone calcification patterns on paravalvular regurgitation after transcatheter aortic valve replacement with different next-generation devices. Open Heart 2020;7:e001164. 32393655 10.1136/openhrt-2019-001164 PMC7223472

[10] Ochiai T , Yamanaka F, Shishido K, Moriyama N, Komatsu A, Yokoyama H, et al Impact of high implantation of transcatheter aortic valve on subsequent conduction disturbances and coronary access. JACC Cardiovasc Interv 2023;16:1192–1204. 37225290 10.1016/j.jcin.2023.03.021

[11] Auffret V , Puri R, Urena M, Chamandi C, Rodriguez-Gabella T, Philippon F, et al Conduction disturbances after transcatheter aortic valve replacement: current status and future perspectives. Circulation 2017;136:1049–1069. 28893961 10.1161/CIRCULATIONAHA.117.028352

[12] Ribeiro HB , Webb JG, Makkar RR, Cohen MG, Kapadia SR, Kodali S, et al Predictive factors, management, and clinical outcomes of coronary obstruction following transcatheter aortic valve implantation. J Am Coll Cardiol 2013;62:1552–1562. 23954337 10.1016/j.jacc.2013.07.040

[13] Russo G , Tang G, Sangiorgi G, Pedicino D, Enriquez-Sarano M, Maisano F, et al Lifetime management of aortic stenosis: transcatheter versus surgical treatment for young and low-risk patients. Circ Cardiovasc Interv 2022;15:e012388. 10.1161/CIRCINTERVENTIONS.122.012388 36378737

