# Supplementary material for: High implantation of a balloon-expandable valve above the left ventricular outflow calcification improves the prosthetic valve function without increasing complications: a case series
Source: Eur Heart J Case Rep. 2025 Jan 10;9(1):ytaf007. doi: 10.1093/ehjcr/ytaf007 (PMC11772999; doi:10.1093/ehjcr/ytaf007)
Supplement: ytaf007_Supplementary_Data [file ytaf007_supplementary_data.zip › supplementary_material revise.docx]

**Supplementary materials**

**High implantation of a balloon-expandable valve above the left ventricular outflow calcification improves the prosthetic valve function without increasing complications: a case series**

**Transcatheter Heart Valve sizing**

The choice of the valve size is the same for high implantation techniques as it is for normal implantation and is based on the annulus size on computed tomography. On the other hand, patients with severe left ventricular outflow tract calcification often have high leaflet calcification, and care must be taken regarding the risk of sinus of Valsalva (SOV) injury and coronary occlusion; thus, we consider the SOV size in patients with a borderline-size annulus.

**High implantation technique (Non coronary cusp zero-position deployment) for the balloon-expandable valve**

First, we placed a pigtail catheter at the bottom of the non-coronary cusp in the annular coplanar view. After inserting the balloon-expandable (BE) valve, the perspective angle was slowly swung to the left oblique position to eliminate the anterior–posterior disparity of the BE valve (Figures S1a and S1b). After rapid right ventricular pacing of greater than 180 beats per minute, we slowly inflated and implanted the BE valve such that the lower left stent bottom frame of the BE valve was attached to the lower edge of the non-coronary cusp. Next, we inflated the valve such that the resulting left coronary cusp side of the BE valve was implanted above the left ventricular outflow tract calcification (LVOTC) (Figures S1c and S1d). Furthermore, even if the LVOTC was under any coronary cusp, we implanted the BE valve by swinging the view from the coplanar view to the left oblique to eliminate the transcatheter heart valve (THV) disparity. This view is suitable for THV implantation above the LVOTC because the left coronary cusp is isolated, the bottom of the non-coronary cusp is secured by the placed pigtail catheter, and the bottom of the right coronary cusp is slightly above the non-coronary cusp.

**Figure legends**

Supplementary figure 1: The angle and depth of deploying the valves

a: coplanar view, b: left oblique position, c, d: deploying the valve was performed in two stages.
